# The Alzheimer's Biomarker Consortium–Down Syndrome (ABC‐DS): A 10‐year report

**DOI:** 10.1002/alz.70294

**Published:** 2025-05-15

**Authors:** Benjamin L. Handen, Mark Mapstone, Sigan Hartley, Howard Andrews, Brad Christian, Joseph H. Lee, Dana Tudorascu, Christy Hom, Beau M. Ances, Shahid Zaman, Sharon Krinsky‐McHale, Adam M. Brickman, H. Diana Rosas, Annie Cohen, Melissa Petersen, Sid O'Bryant, Jordan P. Harp, Frederick Schmitt, Lauren Ptomey, Jeffrey Burns, Ira T. Lott, Florence Lai, Wayne Silverman, Charles Laymon, Elizabeth Head, the Alzheimer's Biomarker Consortium – Down Syndrome (ABC‐DS)

**Affiliations:** ^1^ University of Pittsburgh Department of Psychiatry Pittsburgh Pennsylvania USA; ^2^ University of California Irvine Department of Neurology Irvine California USA; ^3^ University of Wisconsin Madison Waisman Center Madison Wisconsin USA; ^4^ Columbia University Irving Medical Center Taub Institute for Research on Alzheimer's Disease and the Aging Brain New York New York USA; ^5^ Columbia University Vagelos College of Physicians and Surgeons Taub Institute for Research on Alzheimer's Disease and the Aging Brain, Department of Neurology New York New York USA; ^6^ Irvine University School of Medicine, of California Department of Psychiatry and Human Behavior Orange California USA; ^7^ Washington University School of Medicine in St. Louis, Box 8111 St. Louis Missouri USA; ^8^ University of Cambridge School of Clinical Medicine Department of Psychiatry Forvie Site, Robinson Way Cambridge UK; ^9^ NYS Institute for Basic Research in Developmental Disabilities Department of Psychology Staten Island New York USA; ^10^ Massachusetts General Hospital Departments of Neurology and Radiology Harvard Medical School Charlestown Massachusetts USA; ^11^ University of North Texas Health Science Center Department of Family Medicine Fort Worth Texas USA; ^12^ University of Kentucky College of Medicine Kentucky Neuroscience Institute & Sanders‐Brown Center on Aging Lexington Kentucky USA; ^13^ University of Kansas Medical Center Kansas city Kansas USA; ^14^ University of California Irvine School of Medicine Department of Pediatrics Orange California USA; ^15^ Massachusetts General Hospital Department of Neurology Harvard Medical School Charlestown Massachusetts USA; ^16^ University of California Irvine Department of Pathology, 1261 Gillespie Neuroscience Facility Irvine California USA

**Keywords:** ABC‐DS, Alzheimer's disease, dementia, Down syndrome

## Abstract

**INTRODUCTION:**

Virtually all adults with Down syndrome (DS) will accumulate the neuropathologies associated with Alzheimer's disease (AD) by age 40, with the majority having a clinical dementia diagnosis by their middle 50s.

**METHODS:**

This paper complements a 2020 publication describing the Alzheimer's Biomarker Consortium–Down Syndrome (ABC‐DS) methodology by highlighting protocol changes since initial funding in 2015. It describes available clinical, neuropsychological, neuroimaging, and biofluid data and bio‐specimen repository. Ten years of accomplishments are summarized.

**RESULTS:**

Over 500 adults with DS and 59 sibling controls have been enrolled since 2015 with nearly 800 follow‐up visits. More than 900 magnetic resonance imaging (MRI), 800 amyloid positron emission tomography (PET), and 600 tau PET scans have been conducted; multiple omics data have been generated using over 1100 blood and 100 cerebrospinal fluid (CSF) samples.

**DISCUSSION:**

ABC‐DS is the largest U.S.‐based, multi‐site (including the United Kingdom and Puerto Rico), longitudinal biomarker initiative to target adults with DS at risk for AD.

**Highlights:**

- The Alzheimer's Biomarker Consortium—Down Syndrome (ABC‐DS) is entering its 10th year.
- Over 500 adults with Down syndrome (DS) and 59 sibling controls have been enrolled.
- More than 900 magnetic resonance imaging (MRI), 800 amyloid positron emission tomography (PET), and 600 tau PET scans have been conducted.
- Multiple omics data have been generated using over 1100 blood and 100 cerebrospinal fluid (CSF) samples.
- It is positioned to continue to make substantial contributions to the DS field.

## 1 BACKGROUND

Down syndrome (DS) was initially described in the first half of the 19th century by two French physicians, Dr Jean‐Etienne Dominique Esquirol and Dr Edouard Seguin. The syndrome was named after Dr John Langdon Down, a British physician who subsequently provided the most complete description of DS by documenting the phenotypical features of the disorder in his 1887 book, “Mental affections of childhood and youth.” However, it was not until nearly 75 years later (in 1959) that the genetic basis for DS was discovered, the triplication of the 21st chromosome, by the French geneticist Jérôme Jean Louis Marie Lejeune and the French cardiologist and researcher, Marthe Gautier. As the life expectancy of individuals with DS has more than quadrupled since the 1950s, a range of co‐occurring medical conditions have been noted in adulthood. Perhaps the most concerning has been the finding that over 90% of adults with DS develop Alzheimer's disease (AD, DS‐AD) by their early to mid‐60s.^1^, ^2^ In fact, most adults with DS will have the neuropathologies characteristic of AD by the time they reach their early to mid‐40s that anticipates further progression some years or decades later.^3^, ^4^, ^5^, ^6^

DS‐AD is likely the single greatest impediment to extending life expectancy of adults with DS beyond their 60s.^7^ While significant gains have been made during the past 20–30 years toward increasing understanding of the causes, risk factors, and potential treatment of sporadic AD, the DS population has generally been excluded from this work.^8^ Important early work in DS‐AD was conducted by a small number of eminent researchers beginning in the late 1980s.^9^ Subsequent funding opportunities for this area of research began to increase significantly approximately 15 years ago with a number of parallel research efforts in Europe and the United States, including work by a consortium of European Universities and hospitals (Horizon21), efforts funded by the LuMind Foundation (LIFE‐DSR), and the NIA/NICHD funded multicenter study of biomarkers of AD in adults with DS (the Alzheimer's Biomarker Consortium – Down Syndrome [ABC‐DS]). In addition, in 2018, the INCLUDE Project (INvestigation of Co‐occurring conditions across the Lifespan to Understand Down syndrome) was launched to support a new trans‐National Institutes of Health (NIH) research initiative on critical health and quality‐of‐life needs for individuals with DS. The National Institute on Aging (NIA) has also recently funded the development of a “trial ready cohort” of adults with DS (TRC‐DS; R61 AG066543) and several active efforts to initiate large‐scale AD prevention trials in DS in the United States and Europe are now underway.

This paper complements a 2020 publication^10^ describing the ABC‐DS methodology by highlighting protocol changes since the consortium was initially funded in 2015. It describes currently available clinical, neuropsychological, neuroimaging, neuropathological, and omic data as well as the ABC‐DS bio‐specimen repository. We also summarize accomplishments over the past 10 years. It is hoped that this paper will serve as a stimulus for investigators outside of ABC‐DS, especially early career researchers and trainees, to leverage this valuable, longitudinal database and associated bio‐specimens.

## 2 METHODS

### 2.1 ABC‐DS history and aims

There are currently close to a quarter of a million individuals with DS in the United States alone and over five million world‐wide.^11^ Recognizing the growing significance of DS‐AD, the NIA and National Institute of Child Health and Human Development (NICHD) announced a Request for Application (RFA) in 2014 for a longitudinal investigation of biomarkers of AD among adults with DS with the eventual goal of discovering targets for future clinical prevention trials. In 2015, the ABC‐DS study was funded (ABC‐DS 2015) as two linked projects: “Neurodegeneration in Aging Down Syndrome (NiAD)” and “Alzheimer's Disease in Down Syndrome (ADDS).” These two projects included the efforts of six university‐based programs in the United States and one in the United Kingdom. Table 1 lists the six initial clinical performance sites along with four newer sites and ten secondary research sites that provide a range of important support services (e.g., quality control [QC] for MRI and PET scans, data storage, blood banking). All of the initial ABC‐DS sites had either well‐established research legacy cohorts of adults with DS or adult medical clinics that specifically served the needs of this population (or both).

**RESEARCH IN CONTEXT**

1. **Systematic review**: This paper complements a 2020 publication describing the Alzheimer's Biomarker Consortium–Down Syndrome (ABC‐DS) methodology by highlighting protocol changes since the consortium was initially funded in 2015. It describes currently available clinical, neuropsychological, neuroimaging, neuropathological, and omic data as well as the ABC‐DS bio‐specimen repository. We also summarize accomplishments over the past 10 years.
2. **Interpretation**: The ABC‐DS has enrolled over 500 adults with DS and 59 sibling controls since 2015 with nearly 800 follow‐up visits. More than 900 magnetic resonance imaging (MRI), 800 amyloid positron emission tomography (PET), and 600 tau PET scans have been conducted; multiple omics data have been generated using over 1100 blood and 100 cerebrospinal fluid (CSF) samples.
3. **Future directions**: The ABC‐DS continues to follow this large cohort of at‐risk adults with DS and to enroll new participants. The consortium has already had a significant impact on the design and selection of assessment tools to be used in many of the Alzheimer's disease (AD) prevention trials for the DS population. It is positioned to continue to make substantial contributions to our understanding of AD in adults with DS and the identification of biomarkers of preclinical AD progression.

**TABLE 1:** ABC‐DS sites 2015–2026.

Site	PIs	Role	Year joined
University of Pittsburgh	Handen/Cohen/Klunk^*^	Clinical performance	2015
Columbia University/Institute for Basic Research in DD	Lee/Krinsky‐McHale/Schupf^*^	Clinical performance	2015
University of Wisconsin, Madison	Christian/Hartley	Clinical performance	2015
Mass. General Hospital	Rosas/Lai	Clinical performance	2015
University of California Irvine	Lott/Hom	Clinical performance	2015
University of Cambridge	Zaman	Clinical performance	2015
Washington Un	Ances	Clinical performance	2019
University of Kentucky	Schmitt/Harp	Clinical performance	2020
University Kansas Medical Center	Ptomey/Burns	Clinical performance	2023
University of Puerto Rico	Salas/Pagán	Clinical performance	2024
National Centralized Repository for Alzheimer's Disease and Related Dementias (NCRAD)	Foroud	Bio‐banking and *APOE* determination	2015
Alzheimer's Therapeutic Research Institute (ATRI)	Aisen/Rafii	Data management	2015
The Laboratory of Neuro Imaging (LONI)	Toga	Data hosting	2015
Mayo Clinic	Jack	PET QC	2015
University of Michigan	Koeppe	MRI QC	2015
University of North Texas Health Science Center (UNTHSC)	O'Bryant/Petersen	Proteomics assay analysis	2015
Georgetown University	Cheema	Metabolomics assay analysis	2015
Wash U CSF Center	Fagan^†^/Schindler	CSF analysis and storage	2019
Johns Hopkins University	Wang	Statistical consultation	2015
Indiana University Lab	NA	Clinical lab analyses	2020

*Note*. The Banner Institute left in 2018 after recruiting a small number of participants (PI: Sabbagh).

Abbreviations: ABC‐DS, Alzheimer's Biomarker Consortium–Down Syndrome; PI, principal investigator.

* 2015—2020.

^†^ 2015–2023.

ABC‐DS was extended in 2020 (ABC‐DS 2020) for another 5 years, now as a single grant that integrated the NiAD and ADDS more formally and expanded the program to eight clinical performance sites plus additional supporting resources with over 90 co‐investigators. It is led by four multiple principal investigators (MPIs) (Benjamin Handen, Elizabeth Head, Bradley Christian, and Mark Mapstone) and a senior advisory board (Wayne Silverman, Ira Lott, Nicole Schupf, and William Klunk) comprising past co‐PIs of ADDS and NiAD who elected to transition to an emeritus status. Subsequently, two additional clinical performance sites were added (the University of Kansas Medical Center in 2024 and the University of Puerto Rico in 2025). The 2020 grant also led to a revised network structure with a set of specific cores and projects. The University of Pittsburgh currently serves as the administrative coordinating center for ABC‐DS. There are seven cores: Administrative, Clinical, Omics, Neuroimaging, ADDORE (Alzheimer's disease/Down syndrome Outreach, Recruitment, and Engagement), BDM (Biostatistics and Data Management), and Neuropathology, along with three projects: Project 1 – To determine whether AD in DS parallels late onset AD within an amyloid (A), tau (T), neurodegeneration (N) – the ATN framework and to identify modifiers of risk of conversion/progression; Project 2 – Seeks to identify genetic modifiers of AD progression in DS; and Project 3 – To develop a precision medicine framework for DS‐AD that could be used to expedite clinical trials. To facilitate the completion of the Project goals, five overarching specific aims were also established:

Aim 1: Establish an organizational infrastructure to follow and comprehensively characterize a clinical cohort of 600 individuals. Using a traditional Core structure for program oversight, recruitment, and deep phenotyping through longitudinal data collection and analysis, data from an expanded cohort of 600 individuals (550 with DS and 50 sibling controls), followed at 16‐month intervals, is being collected, including clinical, cognitive, neuroimaging, and other data in addition to bio‐specimens for omics analyses. A priority is placed on recruiting from racially and ethnically minoritized families for new participants.

Aim 2: Examine biomarkers of DS‐AD progression, expanding upon the ATN framework, and determine if this progression is modified by selected non‐genetic factors. Particular interest is placed on determining if a priori selected factors (e.g., sex, co‐occurring medical conditions, cerebrovascular pathology, or inflammation) are modifiers of ATN in DS.

Aim 3: Examine genetic factors that modify DS‐AD risk. Here, interest is placed on broad genetic contributions using whole genome sequencing (WGS) and integrating findings with other omic modalities and other imaging markers.

Aim 4: Develop precision medicine ready biomarkers for DS‐AD clinical trials. The goal is to provide a hierarchy of informative biomarkers that progress from less‐ to more‐invasive and costly, to guide screening of prospective participants, stratify endophenotypes, and serve as surrogate outcomes for next‐generation DS‐AD clinical trials.

Aim 5: Disseminate data and biospecimens to qualified researchers outside the ABC‐DS to support expanded research. Data and specimen sharing is provided to the broader scientific community through NIH‐funded, public‐facing dissemination nodes hosted by the Laboratory for Neuro Imaging (LONI), the Alzheimer's Therapeutic Research Institute (ATRI), and the National Centralized Repository for Alzheimer's Disease and Related Dementias (NCRAD).

Although the outcome of these aims is beginning to inform models that can predict the trajectory of the disease and its clinical manifestations at the individual level of this genetic form of AD, without a more detailed characterization of known and novel biomarkers, personalization of prognosis and diagnosis will falter.

### 2.2 ABC‐DS procedures

**FIGURE 1:**
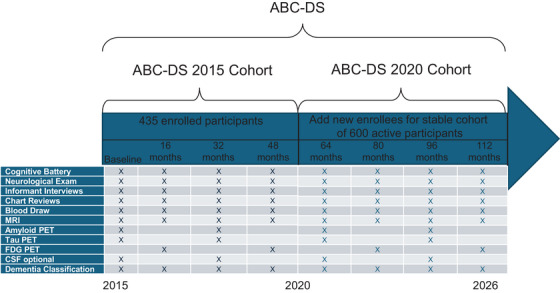
Overview of ABC‐DS procedures at each scheduled study visit. ABC‐DS, Alzheimer's Biomarker Consortium–Down Syndrome.

ABC‐DS is a study of adults with DS (ages 25+) involving a multi‐cohort, longitudinal design to identify cognitive, blood, cerebrospinal fluid (CSF), and neuroimaging biomarkers for predicting the onset and progression of DS‐AD. Because virtually all people with full trisomy 21 are expected to develop the pathophysiological changes of AD by their earyl to mid‐40's, this longitudinal design provides a unique opportunity to track physiological and cognitive changes through the clinically asymptomatic preclinical to the clinical stages of dementia. Participants are enrolled based on well‐defined inclusion and exclusion criteria and are followed via an in‐person visit to one of the Clinical Performance sites at the time of enrollment (baseline) and approximately every 16 months thereafter until study conclusion. At study entry, most participants (> 77%) had not yet evidenced AD‐related clinical symptoms. The study also includes a small number of healthy, neuro‐typical sibling controls that allows us to further isolate contributions to dementia risk and progression through the shared genetic variance with their DS sibling participants. Procedures performed at each assessment visit include a physical/neurological examination, cognitive assessment battery, informant/caregiver questionnaires, MRI and PET scans (amyloid, tau, and fluorodeoxyglucose [FDG]), peripheral blood draw, and optional lumbar puncture (LP) for collection of a small sample of CSF (see Figure 1). These procedures are used to characterize and document change in cognitive/behavioral and functional states in order to classify participants into groups based on clinical status (cognitively stable, mild cognitive impairment [MCI], dementia, undetermined). Clinical classifications are based on individual consensus conferences involving expert DS clinicians, study coordinators, and highly trained and experienced staff who are familiar with each participant. This team is blind to neuroimaging, genetics, and omics data for participants discussed at conference but has access to clinical blood lab panel results (e.g., thyroid stimulating hormone [TSH], glucose, and cholesterol levels). Other outcomes are collected for their potential as biomarkers of current and future clinical AD status or to identify potential modifiers of AD progression. Finally, a brain donation program is in place which allows researchers to connect in vivo clinical and cognitive data, omics, and neuroimaging measures with histopathology and other *post mortem* outcomes in a clinicopathological framework.

The goal of ABC‐DS 2020 is to enroll and provide deep phenotyping of 550 adults with DS and 50 sibling controls (an increase from the original ABC‐DS 2015 cohort of 350 adults with DS and 50 sibling controls). It was anticipated that at least 70% of ABC‐DS 2015 enrollees would carry‐over to ABC‐DS 2020. Adding new participants would allow for increased efforts to improve the diversity and representation of the cohort and to replace individuals lost to follow‐up.

The primary biomarkers of interest have remained fairly consistent since ABC‐DS began. For example, the PET neuroimaging biomarkers are unchanged and include amyloid PET and tau PET scans at baseline and Follow‐up visit 2 and an FDG PET scan at Follow‐up visit 1. MRI scans are conducted at each visit, consisting of the sequences defined for the ADNI4 study: T1‐weighted, T2‐weighted fluid attenuated inversion recovery (FLAIR), susceptibility weighted imaging (SWI), arterial spin labeling (ASL), diffusion tensor imaging (DTI), and resting state functional MRI (rs‐fMRI). The scanning procedures are generally well‐tolerated, and sedation is never used. Participants are encouraged to remain in the MRI scanner for as long as they are able. Fluid biomarkers from blood include traditional AD proteomic biomarkers such as Aβ_40,_ Aβ_42_, tau, ptau181, and others as described in Handen et al. 2020, as well as emerging biomarkers, including ptau217, NT1‐tau, neurofilament light chain (NfL), and glial fibrillary acidic protein (GFAP). Metabolomic biomarkers that index specific biochemical pathways, including glucose metabolism, oxidative stress, cholesterol metabolism, inflammation, and amino acid metabolism among others, are also obtained. For those willing to undergo an optional LP, standard AD‐related proteins including Aβ_40_, Aβ_42_, tau, and ptau181 are obtained in addition to emerging biomarkers such as YKL‐40, NfL, and VILIP‐1. Genomic data include karyotyping, genome‐wide microarray single nucleotide polymorphism (SNP) data (typically called genome‐wide association studies [GWAS] data) and WGS data are available. These genomic data allow investigators to examine the entire human genome as well as known AD candidate genes, such as *APP*, apolipoprotein E (*APOE)*, *SOD1*, *S100β*, and SORL1 to better understand the genotype‐phenotype relationship in individuals with DS.

Data are locked every 16–24 months, and after rigorous quality assurance and quality control procedures, are hosted on the LONI platform. Each data lock is referred to as a “Freeze.” In this paper, we summarize available data through “Freeze 4,” which occurred on September 15, 2023. Currently available data on the LONI platform include cognitive test results, caregiver questionnaires, health history, scans (including raw images and outcome variables), proteomics results, metabolomics results, selected genetics results (e.g., *APOE*, karyotyping, ABC‐DS 2015 GWAS), CSF analyses, and clinical lab findings. Neuropathology data being collected include *post mortem* MRI and final neuropathology reports using the National Alzheimer disease Coordinating Center Neuropathology forms that stages AD pathologies (e.g., Braak stage, Thal stage, neuritic plaque stage, or ABC), cerebrovascular pathologies, and the presence and location of other co‐pathologies (e.g., TDP‐43, Lewy bodies, hippocampal sclerosis)(https://naccdata.org/data‐collection/forms‐documentation/np‐11). ABC‐DS is also using whole slide imaging and digital pathology to acquire unique measures of beta‐amyloid plaque and tau neurofibrillary tangle “loads” with new measures being added (e.g., microglial loads, phenotype, and markers of astrogliosis).

The cognitive assessment battery underwent a number of changes in the ABC‐DS 2020 protocol (relative to ABC‐DS 2015), with the goal of shortening the length of administration. The current battery requires 90–120 min to complete. The caregiver questionnaires remain essentially unchanged from the ABC‐DS 2015 protocol, apart from the elimination of the American Association on Mental Deficiency Adaptive Behavior Scale.^12^ Table 2 lists the ABC‐DS cognitive and caregiver measures that are available in the current “Freeze 4” data base (available on LONI). It should be noted that sibling controls undergo the collection of the same biomarkers as the DS adults (except for karyotyping), but no caregiver measures are obtained, and the cognitive assessment is limited to the Montreal Cognitive Assessment (MoCA)^13^ and the AD8 Dementia Screening Interview.^14^

**TABLE 2:** Cognitive and caregiver measures.

Domain	Task
Mental status	Down Syndrome Mental Status Examination^15^
	Rapid Assessment for Developmental Disabilities (RADD)^16^
Dementia symptoms	Dementia Questionnaire for People with Learning Disabilities (DLD)^17^
	NTG‐Early Detection Screen for Dementia (NTG‐EDSD)^18^
Functional abilities	Vineland Adaptive Behavior Scale, 3rd Edition^19^ (cycle 1 only)
Language/cognitive	Categorical/Verbal Fluency^20^, ^21^
	KBIT‐2^22^ (only at cycle 1 visit)
Visuospatial construction	Block Design and Extended Block Design^15^, ^23^
	Beery Buktenica Developmental Test of Visual Motor Integration^24^
New learning and memory	Modified Cued Recall Test^25^
Neuropsychiatric symptoms/behavior problems	Neuropsychiatric Inventory (NPI)^26^
	Reiss Screen for Maladaptive Behavior^27^
Executive processing and speed	The Stroop Cats and Dogs Task^28^
	The Purdue Pegboard^29^
	The Cancellation Task^30^
Gait	Tinetti Assessment Tool: Gait^31^
Health status and life events	Demographic health questionnaire
	Life Stressors Index [Seltzer G, personal communication, 2002]
	Physical and clinical labs

Finally, all data collected in the study are made available, without embargo, to qualified outside investigators through the NIH‐sponsored data repositories at LONI at the University of Southern California. Bio‐specimens are stored at the bio‐specimen repository (NCRAD) at the University of Indiana, the CSF Laboratory at Washington University (Wash U CSF Lab), and the Brain Tissue Repository at the University of California Irvine. As these samples are extremely important and limited, requests undergo a much higher level of review (see Data and BioSample Availability and Directions for Accessing Data and/or Biosamples below).

### 2.3 Inclusion/Exclusion criteria

There are three groups of study participants who have been enrolled in ABC‐DS 2020: (1) Individuals with DS who were previously enrolled in ABC‐DS 2015, (2) individuals with DS who are new to ABC‐DS, and (3) sibling controls (both those previously enrolled in ABC‐DS 2015 and those new to the study). There are no inclusion/exclusion criteria for “carry‐over participants” (those previously enrolled in ABC‐DS 2015), as it was felt to be important to continue to follow all prior enrollees, regardless of current dementia status. Therefore, the following inclusion/exclusion criteria apply only to individuals new to ABC‐DS 2020:

Inclusion criteria for participants with DS:

1. Age 25 years of age and older;
2. Estimated mental age of ≥ 3.0;
3. Karyotype of full trisomy 21, partial trisomy or mosaic DS (as confirmed by updated karyotyping or medical record review);
4. English speaker from an early age;
5. A reliable study partner* capable of providing information about clinical symptoms and history;
6. Agreement of study partner and clinicians that the participant is able to cooperate with the protocol tasks;
7. Adequate visual and auditory acuity to complete neuropsychological testing;
8. Informed consent of participants or informed consent by a legally authorized representative and participant assent.

Inclusion criteria for sibling controls:

1. Age 25 years of age and older;
2. Willingness to cooperate with the protocol tasks;
3. Full or half‐sibling of individual with DS who is a study participant;
4. Adequate visual and auditory acuity to complete assessments;
5. Informed consent.

Exclusion criteria for participants with DS:

1. Diagnosis of dementia;
2. Any significant disease or unstable medical/psychiatric condition that could affect neuropsychological testing (i.e., unstable cardiac problems, chronic renal failure, chronic hepatic disease);
3. Participants in whom MRI is contraindicated including, but not limited to, those with a pacemaker, presence of metallic fragments near the eyes or spinal cord, cochlear implant, claustrophobia, or problems with blood draws;
4. Pregnancy, breast feeding;
5. Presence of motor or sensory impairments severe enough to interfere with neuropsychological testing.

Exclusion criteria for sibling controls:

1. A diagnosis of MCI, AD, or other type of dementia;
2. Any significant disease or unstable medical condition that could affect neuropsychological testing (i.e., unstable cardiac problems, chronic renal failure, chronic hepatic disease, severe pulmonary disease);
3. Participants in whom MRI is contraindicated including, but not limited to, those with a pacemaker, presence of metallic fragments near the eyes or spinal cord, cochlear implant, or claustrophobia (dental fillings do not present a risk for MRI);

5. Pregnancy, breast‐feeding.

*Study partners: A family member, caretaker, or other adult who knows the participant with DS well, including their health history, has regular contact with them (e.g., once a week for an extended period) and can describe their functioning.

## 3 RESULTS

### 3.1 Participants

Since its inception in 2015, ABC‐DS has undergone four data locks, with the most recent occurring in September 15, 2023. Table 3 summarizes the demographics of the participants with DS and sibling controls at their baseline visit, including sex, age, karyotype, *APOE* status, co‐occurring medical conditions, and clinical AD status at the first consensus conference.

**TABLE 3:** Baseline demographics (as of 9/15/23).

	DS	SIBS
Variables	*N* (%)	*N* (%)
Sex		
Male	272 (54%)	13 (22%)
Female	231 (46%)	46 (78%)
Age (years)		
25–34	104 (21%)	14 (24%)
35–44	171 (34%)	19 (32%)
45–54	147 (29%)	13 (22%)
55–64	72 (14%)	10 (17%)
65+	9 (2%)	3 (5%)
Race		
White	459 (91%)	59 (100%)
Black	7 (1%)	0 (0%)
Hispanic	24 (5%)	0 (0%)
Other/missing	13 (3%)	0 (0%)
Karyotype		
Non‐trisomic	0 (0%)	59 (100%)
Full trisomy	440 (87%)	0 (0%)
Mosaic	19 (4%)	0 (0%)
Translocation	21 (4%)	0 (0%)
Missing	23 (5%)	0 (0%)
Status		
Cognitively stable	369 (73%)	59 (100%)
MCI‐DS	59 (12%)	0 (0%)
Dementia	54 (11%)	0 (0%)
Undetermined	21 (4%)	0 (0%)
Co‐occurring		
Stroke	7 (2%)	0 (0%)
Diabetes	19 (5%)	0 (0%)
Hyperlipidemia	113 (31%)	5 (8.5%)
Hypothyroidism	221 (60%)	5 (8.5%)
Seizures	34 (9%)	2 (3.4%)
Obesity	179 (48%)	Not Available
*APOE*		
Any ε4 allele	117 (23%)	16 (27%)
ε2 or ε3 allele	366 (73%)	41 (70%)
Missing	20 (4%)	2 (3%)

Abbreviations: *APOE*, apolipoprotein E; DS, Down syndrome; MCI‐DS, mild cognitive impairment‐Down syndrome; SIBS, siblings.

Of the 503 participants with DS enrolled in the 2015 and/or 2020 ABC‐DS studies, 59 (11.7%) were diagnosed with MCI and 54 (10.7%) were diagnosed with AD at baseline (note that individuals with MCI or AD were included in ABC‐DS 2015). The remaining 390 individuals were determined to be either cognitively stable (CS; *n* = 369) or Unable to be Determined due to evidence of a complicating medical concern unrelated to DS‐AD or a recent traumatic life event (*n* = 21). Among those who completed at least one follow‐up visit (*n* = 346), and adjusting for the number of continuing individuals with an MCI or AD diagnosis at each follow‐up evaluation, 16.11% (*n* = 39) progressed from CS to MCI and 18.02% (*n* = 53) progressed from CS to AD or from MCI to AD. All sibling controls have remained cognitively stable.

Of the 562 individuals enrolled in ABC‐DS 2020 as of September 15, 2023, approximately 50% had been previously enrolled in ABC‐DS 2015. However, due to delays resulting from Covid, prior ABC‐DS 2015 participants continued to be enrolled in ABC‐DS 2020 well after this date. Among the reasons that participants have been lost to follow‐up include death (13 to date, mostly due to complications associated with AD), site closure (for participants from the Banner Institute), and illness (e.g., advanced dementia, medical issues). Although not a requirement for participation in ABC‐DS, 12 participants have provided brain donations. In these cases, arrangements were made by the brain donation coordinator and typically both formalin fixed paraffin embedded (FFPE) and frozen samples were acquired to support research efforts into the neurobiology of aging and AD in DS.

### 3.2 The ABC‐DS data corpus

The ABC‐DS performance sites will continue to actively enroll new participants with DS (until reaching an N of 600) while also scheduling those already enrolled to return for subsequent follow‐up visits. The consortium was recently granted approval for a 12‐month extension of Year 04, which will result in a 6‐year study ending August 31, 2026. The latest data freeze (“Freeze 5”) occurred in the fall of 2024 with a subsequent data freeze planned for the winter of 2026. As a result, additional data and bio‐samples should be available to external investigators within 6–7 months of each scheduled data freeze. Below is a summary of available data up to the most recent data freeze held September 2023 (comprising data collected from July 2015 through September 2023).

Table 4 summarizes the number of cognitive assessments, study partner questionnaires, and blood/CSF/neuroimaging biomarkers obtained for participants with DS and sibling controls at baseline and each subsequent follow‐up visit. Over 500 DS adults with DS and 59 sibling controls have enrolled in ABC‐DS since 2015. Almost 350 enrollees with DS have returned for at least one follow‐up visit, providing investigators with the opportunity to address several significant questions related to long‐term changes in areas of cognition, functioning, and biomarkers in the DS population.

**TABLE 4:** Cognitive assessments, caregiver questionnaires, and biomarker collection.

Parameter	No. with a baseline	No. with 1 FU	No. with 2 FU	No. with 3 FU	No. with 4 FU	Total
Caregiver Questionnaires DS	503	346	283	138	4	1274
Cognitive Assessments DS	501	339	280	138	4	1262
**MRI**						
T1 Struct						
DS	426	230	133	35	–	824
SIBS	59	19	5	–	–	83
T2 FLAIR						
DS	412	211	118	24	–	765
SIBS	57	18	3	–	–	78
T2 Star						
DS	327	128	51	–	–	506
SIBS	53	17	1	–	–	71
ASL						
DS	277	97	41	–	–	415
SIBS	55	16	3	–	–	74
DTI						
DS	395	193	108	21	–	717
SIBS	56	18	3	–	–	77
T2 FSE						
DS	276	93	37	–	–	406
SIBS	58	15	3	–	–	76
SWI						
DS	165	74	29	–	–	268
SIBS	15	5	–	–	–	20
fMRI_1						
DS	300	158	70	–	–	528
SIBS	49	16	–	–	–	65
fMRI_2						
DS	88	–	–	–	–	88
SIBS	9	–	–	–	–	9
**PET**						
Amyloid PET						
DS	425	193	106	14	–	738
SIBS	53	17	4	–	–	74
Tau PET						
DS	300	158	66	–	–	524
SIBS	54	17	5	–	–	76
FDG PET						
DS	97	–	–	–	–	97
SIBS	25	–	–	–	–	25
**OMICS**						
Blood collection						
DS	501	325	244	107	–	1,177
SIBS	59	20	–	–	–	79
CSF collection						
DS	72	21	7	–	–	100
SIBS	7	–	–	–	–	7

Abbreviations: ASL, arterial spin labeling; CSF, cerebrospinal fluid; DS, Down syndrome; DTI, diffusion tensor imaging; DWI, diffusion weighted imaging; FDG, fluorodeoxyglucose; FLAIR, fluid attenuated inversion recovery; fMRI, functional magnetic resonance imaging; FSE, fast spin echo; FU, follow‐up; PET, positron emission tomography; SIBS, siblings; SWI, susceptibility weighted imaging.

#### 3.2.1 Cognitive and study partner data

Over 1250 cognitive assessments have been conducted and a similar number of study partner questionnaires obtained since 2015 (see Table 4). Over 60% of the data collected has occurred at follow‐up visits, providing considerable opportunities for the examination of longitudinal changes in cognition and functioning. For example, 280 DS individuals have completed a baseline and at least two subsequent follow‐up cognitive assessments. It should be noted that during the coronavirus disease 2019 (COVID‐19) pandemic (2020–2022), many of the in‐person visits could not be scheduled and only study partner data was obtained. In addition, as individuals with DS developed more significant AD symptoms, travel to study sites could be challenging, and administration of cognitive assessments more difficult. In such cases, only study partner data continued to be collected or an abbreviated cognitive assessment conducted.

#### 3.2.2 Neuroimaging data

As shown on the Schedule of Assessments (Figure 1), most PET scans are completed at every other study visit for the amyloid and tau measures (at baseline and at follow‐up 2). As shown on Table 4, approximately 80% of ABC‐DS participants have undergone at least one amyloid PET scan while just under 60% have undergone at least one tau PET scan; approximately 85% of participants have been able to cooperate for at least one T1‐weighted MRI sequence. Relatively high rates of successful FLAIR, SWI, fMRI, ASL, and DTI MRI sequences have also been obtained. Finally, amyloid/tau/FDG PET scans and the primary MRI sequences were successfully conducted with the majority of sibling controls.

#### 3.2.3 Proteomics/metabolomics data from plasma or CSF

Blood draws occurred at each baseline and all follow‐up visits while an optional LP was conducted at every other follow‐up visit (only for willing participants). The rate of successful blood draws has been consistently high (above 90%), resulting in longitudinal proteomics/metabolomics assay results being available for hundreds of participants across multiple time points. This also allows for comparison of plasma‐based biomarkers with both neuroimaging and cognitive data. As anticipated, only a small number of participants agreed to undergo an LP procedure for the collection of CSF; a quarter of this group returned for at least one follow‐up LP as of the writing of this manuscript.

#### 3.2.4 Genetics data

Genome‐wide microarray data have been generated for 418 study participants (specifically, 375 adults with DS and 59 sibling controls). These genomic data are currently available on the Image & Data Archive (IDA; https://ida.loni.usc.edu). In addition, WGS data on 534 participants (472 adults with DS and 55 sibling controls) are being generated at the time of manuscript writing. *APOE* genotype data are available for all study participants. Lastly, karyotyping information is available for all DS study participants from either medical records or from karyotyping, allowing investigators to examine the effects of gene dosage on phenotypic expressions of biomarkers or clinical outcomes.

#### 3.2.5 Neuropathological data

Families have graciously donated 12 brains from ABC‐DS participants who have died (allowing for direct comparison of premorbid biomarker and clinical/cognitive findings with neuropathological findings). The Neuropathology core provides final neuropathology reports to families and also submits forms capturing neuropathology data (e.g., information on the extent of beta‐amyloid plaque and neurofibrillary tangle accumulation, cerebrovascular pathology, Lewy bodies, TDP‐43, and other descriptors) to the National Alzheimer disease Coordinating Center for inclusion in analyses conducted by international studies. Each case is also thoroughly discussed for in depth characterization of all findings. Studies of brain tissues from our donors have led to insights into AD pathogenesis, cerebrovascular pathology and *post mortem* neuroimaging correlates.^32^, ^33^ Brain tissue remains available to all qualified investigators through our biospecimen request portal.

### 3.3 Data and BioSample availability and directions for accessing data and/or biosamples

The LONI platform houses the current ABC‐DS database, which is also available to all qualified investigators. There are no embargo policies, as data is made available on LONI to both internal and external investigators at the same time. However, some data sets (e.g., cognitive assessment results) may be available before other findings (e.g., genetics). Included are data dictionaries and written descriptions of methods for the various data collection procedures. The primary data set is a combined ABC‐DS 2015/ABC‐DS 2020 file comprising participant demographic information, medical history, medical/neurological examination, caregiver questionnaires, and cognitive assessments. Separate data files have been established for participants with DS and sibling controls. In addition to the primary data set, the raw (i.e., no‐preprocessing) image files of the MRI, amyloid, tau, and FDG PET scans are available for all images that have passed the ADNI‐defined QC process. Derived outcomes for each imaging modality (e.g., amyloid centiloids, tau standardized uptake value ratio (SUVR), regional cortical thickness and volumetry, white matter hyperintensities, microbleeds) are also made available on LONI following scheduled intervals of data release. Raw proteomic and metabolomic data for all analyses are posted along with the results of the ABC‐DS 2015 GWAS. A file with key CSF analyses results is also available. In addition to data generated by ABC‐DS investigators, results generated by secondary analyses conducted by both internal and external investigators are available. For example, if an investigator conducts an assay for a novel plasma‐based biomarker using ABC‐DS samples, they are obligated to submit those results to be hosted on LONI. All data files contain common participant indentifications (IDs) that allow investigators to combine data sets and to easily determine which visit data collection or analyses results occurred. Finally, ABC‐DS data are also shared with the INCLUDE DCC (https://includedcc.org), allowing for viewing ABC‐DS metadata.

The consortium maintains stored blood samples from over 500 adults with DS and 59 sibling controls at NCRAD. In addition, CSF samples are held at the Washington University CSF Center. Requests for ABC‐DS data and/or biospecimens are completed on a Qualtrics‐based website:  ABC‐DS Request Form (qualtrics.com).  A brief request also needs to be completed on the LONI website IDA (usc.edu) and includes the signing of a data agreement to adhere to protecting the deidentification status of the data, complying with acknowledgements and use and disclosure of ABC‐DS data. ABC‐DS maintains a Committee on Publication and Ancillary Studies (CPAS) that reviews all internal and external requests for data. The Committee holds monthly meetings and reviews each proposed study's hypotheses, variables of interest, and planned statistical methods.  An important role of this review process is to limit redundancy in proposed analyses and publications. In cases where two groups of investigators appear to be working in very similar areas, the committee suggests collaboration between the groups. Requests for biospecimens (fluids and/or tissue), once approved by the CPAS, are referred to the Bio‐Specimens Sharing Committee (BSC), which reviews requests for plasma, serum, DNA, CSF, and *post mortem* tissue.  As samples are an extremely valuable and finite resource, these requests undergo a much higher level of review. To date (as of February 27, 2025), there have been 412 data requests, of which 192 (46.6%) were from external investigators. Of the nine approved bio‐sample requests, five have been from investigators outside of ABC‐DS.

### 3.4 ABC‐DS productivity

Currently (as of February 27, 2025) over 200 peer‐reviewed research papers (including a 2020 special issue of *Alzheimer's & Dementia: Diagnosis, Assessment & Disease Monitoring*), a book, 10 book chapters, and hundreds of conference presentations and posters have been published or presented using ABC‐DS data. In addition, with 192 data requests from external investigators, a number of papers have been published or are being prepared involving ABC‐DS data. During the past 10 years, dozens of doctoral and post‐doctoral students have been supported in fields including genetics, biostatistics, psychology, neuroimaging, and neuropathology through ABC‐DS. ABC‐DS data has served as the basis for three NIH‐funded K training awards, “Estimating daily movement for the prevention of Alzheimer's disease in Down syndrome” (K01AG083130; Awardee: Helsel; Mentors: Hartley, Ptomey, Burns), “Toward a neuroscientific understanding of the interaction between Down syndrome and Alzheimer's disease pathology” (K01AG083224; Awardee: Bruno; Mentor: Mapstone), and “Remote assessments for Alzheimer's disease cognitive decline in adults with Down syndrome” (K99AG084738; Awardee: Schworer; Mentors: Hartley, Handen, Petersen), two F training awards, “Weight, Inflammation, and Alzheimer's Disease in Down syndrome” (F31AG085730; Fellow: Fleming; Mentors: Hartley, Christian, Mapstone, Ptomey) and “Synergistic contributions of cerebrovascular disease and neuroinflammation to Alzheimer's disease in adults with Down syndrome” (F31AG090091; Fellow: Edwards: Mentors: Brickman, Head, Wilcock), an Alzheimer's Association Research Fellowship to Promote Diversity (23AARFD‐1022715; Awardee: Aguilar; Mentor: Head), a Jerome Lejeune Foundation grant (Awardee: Aguilar; Mentor: Head), and a Brightfocus Foundation Grant (BFF A2022021F; Awardee: Sordo; Mentor: Head).

In addition, since 2015, ABC‐DS investigators have been awarded over a dozen administrative supplements to provide additional support to the Consortium. Several initial awards were required to harmonize the ABC‐DS 2015 protocol, such as supplements to allow all sites to conduct tau PET scans or to support the consortium's ability to conduct proteomic and metabolomic assays on plasma samples for all participants. Other administrative supplements have been awarded to support the purchase of laboratory equipment. Two supplements in 2019 and 2023 provided the funding to include additional clinical performance sites (Washington University and University of Kansas Medical Center) to the Consortium, while four administrative supplements allowed for the expansion of the ABC‐DS protocol. For example, the MOMs study (Modification of Maternal AD risk in DS) is designed to more closely examine family history of dementia and other parental biomarkers (e.g., plasma amyloid and tau levels) and parental genetic profiles that might have an impact on the emergence of early signs of dementia among ABC‐DS participants. The GAIT Study allows for a more extensive examination of gait (via gait mat technology and movement analysis software) as a potential clinical biomarker for AD and the relationship of changes in gait with other AD biomarkers and changes in cognition. The Lifestyle study provided an opportunity to pilot an examination of the relationship between modifiable factors such as sleep, physical activity level, and social engagement with AD biomarkers and timing of AD symptomology. A neuropsychological outcome study led to the piloting of a set of cognitive measures for individuals with DS functioning in the more severe/profound ranges of intellectual disability (ID). Finally, ABC‐DS investigators have also been able to leverage ABC‐DS resources and data to successfully obtain NIH funding for related investigations, including “Lifestyle and Alzheimer's disease in Down syndrome” (PI Hartley, R01 AG070028), “Cerebrovascular contributions to Alzheimer's disease in adults with Down syndrome” (MPI Brickman/Head, RF1AG079519), and “Heterogeneous genetics effects and mediation in Alzheimer's disease” (PI Wang, R21AG087057‐01A1). Table 5 provides a summary of funded projects (comprising seven administrative supplements and one R01) that demonstrate ABC‐DS's success in leveraging resources and enrolling participants.

**TABLE 5:** Supplements and grants requiring participant enrollment 2015–2025.

Grant	Dates	Proposed *N*	Outcome
Lifestyle and Alzheimer's Disease in Down Syndrome^*^	09/30/18 – 04/30/20	75 time pt. #1 35 time pt. #2	81 enrollees at time point #1 39 enrollees at time point #2
Outcome Measures for Use with Adults with DS and Severe to Profound ID^*^	09/01/19 –08/31/21	30	15 enrollees during 2‐year grant period
Washington University site addition^*^	09/15/19 – 04/30/21	25	14 enrollees during 2‐year grant
Lifestyle and Alzheimer's Disease in Down Syndrome R01 AG070028^†^	09/01/2020 –08/31/2025	140 time pt. #1 140 time pt. #2 140 time pt. #3	149 time pt. #1 91 time pt. #2 58 time pt. #3 as of 3/1/25
MOMs study^*^	09/01/22 –08/31/24	150	152 enrollees
University of Kansas Medical Center site addition^*^	09/01/23 – 08/31/25	20	16 enrollees as of 2/28/25
Gait study^*^	09/01/23 – 08/31/25	120	48 enrollees as of 2/28/25
University of Puerto Rico site addition^*^	09/01/24 – 08/31/25	10	First enrollees slated for 4/1/25

Abbreviations: DS, Down syndrome; ID, intellectual disability; MOMs, Modification of Maternal AD risk in DS study.

* Administrative Supplement.

^†^ R01.

### 3.5 Scientific impact

While it is beyond the scope of this paper to provide a detailed summary of ABC‐DS findings over the past 10 years, it is possible to note some of the significant contributions that have been made by ABC‐DS investigators. In the area of cognition, data from ABC‐DS has established valid measures for detecting MCI and dementia in DS‐AD,^34^, ^35^, ^36^ including eye‐tracking metrics for adults with DS with low premorbid levels of functioning.^37^ This work has also shed light on the profile and sequence of cognitive decline in DS‐AD.^38^, ^39^, ^40^ Declines in episodic memory, which are among the earliest changes detected, have been found to be associated with the presence of both elevated PET Aβ and tau.^41^, ^42^, ^43^ Importantly, work from ABC‐DS investigators indicates a short timeline to symptomatic AD in DS relative to sporadic late onset AD and autosomal dominant AD. Initial memory decline begins after about 3 years of reaching PET Aβ positivity (i.e., CL = 18), aligned with tau deposition across the neocortex. On average, individuals with DS transition to MCI after ∼7 years of PET Aβ positivity and dementia after ∼12 to 13 years.^44^

A number of ABC‐DS papers have also been published on biomarkers and AD progression in DS. For example, DS‐AD has been found to be associated with reduced temporal and parietal cortical thickness, and to be associated with smaller hippocampal and striatal volumes. In addition, cortical thickness can be used to stage DSAD.^45^ Among adults with DS, the striatum has been found to consistently accumulate amyloid earlier than the cortex when measured with PiB^46^. This suggests the striatum is more sensitive to the onset of Pittsburgh Compound B (PiB) PET‐detectable amyloid in DS. Other biomarkers have also been found to play a role in AD progression in DS. For example, we have learned that cerebrovascular pathology is an early phenotype of people with DS^47^ and that neuroinflammation may play a role in driving AD pathogenesis in DS.^48^

Furthermore, even though the general progression of amyloid followed by tau is similar for people with DS and people with ADAD, there are observable yet subtle differences in the spatial distribution, timing, and magnitude of tau PET burden between these two groups. Interestingly, DSAD cortical thickness differences are more extensive and severe in comparison to adults with ADAD.^45^ Conversely, amyloid PET accumulation has been found to be similar between people with ADAD and those with DS when directly compared. ABC‐DS investigators have also focused on the utility of plasma biomarkers. It was recently found that some plasma biomarkers may correlate with cognition in cognitively stable individuals with DS and that lower plasma amyloid beta 42/40 is related to lower visuospatial abilities. Higher plasma total tau and NfL protein levels are associated with lower cognitive performance. Thus, AD plasma biomarkers may provide opportunities to track the earliest stages of dementia in people with DS.^49^ We have also begun placing many of the biomarkers from ABC‐DS within the AT(N) framework, with patterns being parallel to that observed in LOAD. However, biomarker positivity tends to occur earlier and more rapidly in AD progression in DS.^50^ We are observing people with DS who may be resilient to AD neuropathology having relatively intact cognition despite significant numbers of plaques and tangles.  ABC‐DS is now beginning to not only identify risk factors but also protective factors (e.g., *APOE*, lifestyle).^51^

ABC‐DS also allows a unique opportunity to examine the genetic underpinning of phenotypic variations in adults with DS as exemplified by the above observations. To further pinpoint contributing factors for phenotypic variation, our testing of the gene dosage model revealed that mosaicism (having a combination of disomic and trisomic cells) would lower levels of AD biomarkers and confirmed that plasma amyloid biomarkers were significantly lower in mosaicism than in full trisomy^52^. In addition, ABC‐DS investigators have been focusing on the identification of novel genes associated with AD. While their functional roles need to be further characterized, some novel genes include four novel loci associated with amyloid β peptides (*PFKFB3, DLX3/PICART1*; *LINC01941/GYPC* and *PDE4D*) and four loci with tau phenotypes (*TUBAP, CTNND2*, *LSTN2, and JHY*).^53^ Furthermore, ABC‐DS participants, along with other at risk cohorts, showed their relevance in human AD modeling by confirming a novel gene, *MTUS2*, identified from a neurotypical healthy aging cohort.^53^ In addition, our investigators have compared AD progress in DS with that of other at‐risk populations, namely autosomal dominant Alzheimer's disease (ADAD). For example, Gorijala et al^54^ constructed polygenic risk scores (PRS) using the AD genetic risk variants from the neurotypical population, and found that the PRS for AD was not associated with familial early‐onset AD (EOAD) and DS‐AD, but was associated with memory scores in DS, independent of *APOE*.

## 4 DISCUSSION

### 4.1 Challenges

Many of the challenges noted in the Handen et al. 2020 paper^10^ on ABC‐DS 2015 remain to this day. While the ABC‐DS successfully harmonized the original NiAD and ADDS protocols almost 9 years ago, the Consortium faced a new challenge of combining the 2015 data with the 2020 data into a single, seamless database. Another challenge was faced by the neuroimaging core investigators as they grappled with how to best address upgrades in scanner platforms, software, and processing pipelines. Examples include updated versions of the Freesurfer software used for the original MRI analyses and subsequent data releases. The decision was made to reprocess all scans dating back to 2015 using the newer pipeline and software and to make both sets of results available on LONI. In terms of PET scans, ABC‐DS (as well as the entire AD field) has had to give consideration for the derived centiloid results of amyloid PET scans that used different ligands and methods more suitable for longitudinal analyses. We also acknowledge that other radiotracers may be included in the protocol as the project continues, as dictated by distribution and availability. As ABC‐DS has continued to grow, it has recognized the challenge of rapidly integrating new sites into the Consortium. This has involved having systems for training and certifying staff to administer the neuropsychological protocol, instructing teams to conduct consensus conferences, as well as training new site coordinators. Finally, the rapid expansion of fluid biomarker discovery in the neurotypical population at risk for AD has presented challenges for prioritizing these emerging biomarkers in our cohort given the finite supply of biospecimens and the funding needed to pursue these unanticipated opportunities.

Ensuring that ABC‐DS is inclusive of individuals with DS from underserved groups and from rural geographic areas has continued to be a major focus. There have been some recent gains in this area with the inclusion of new sites that have demonstrated success in working with underserved DS communities (e.g., the University of Kansas Medical Center and the University of Puerto Rico) and an increased focus by other ABC‐DS sites on building long‐term and mutually beneficial partnerships with communities that have been historically excluded from research. The ABC‐DS ADDORE Core has developed advisory boards of individuals with DS and their family members to gain feedback on culturally appropriate research and outreach materials and how to make participation meaningful (e.g., result disclosure considerations). The ADDORE team has also worked with community partners to develop a rigorous training for research faculty and staff on community‐based participatory research practices. This training has reached 57 DS investigators and staff to date. There are also other potential biases that could impact the course of AD among adults with DS that could be explored going forward. For example, there is a long history of prejudice against individuals with intellectual disability that contributes to lower self‐esteem, social isolation, limited employment opportunities, and poorer mental health.^55^ In terms of physical health, individuals with intellectual disability often have a greater number of health problems, but less access to health services, resulting in an overall health status that lags behind the general population.^56^ Biases in healthcare provision have historically been known to result in individuals with intellectual disability being denied active or life‐saving treatments.^57^ Finally, epidemiological studies having consistently found significant associations between poverty and the prevalence of intellectual disabilities.^58^

Finally, a significant future challenge will be how to manage the expected large number of active ABC‐DS participants who will enroll in AD prevention and treatment trials. Current plans are to temporarily suspend involvement in the ABC‐DS protocol while an individual participates in a clinical trial and to have them return for ABC‐DS follow‐up visits once the last trial visit has been completed. However, decisions regarding the timeframe for scheduling the return visit may depend upon the particular trial, treatment the individual has received during the trial, and the measures included in the last trial study visit (e.g., PET scans, cognitive assessments). It is thought that ABC‐DS can play a pivotal role in AD prevention trials by providing the opportunity to follow individuals for an extended period of time after trial participation (allowing for examination of the long‐term impact of treatment on AD biomarkers and symptoms).

### 4.2 Future plans

The ABC‐DS Consortium remains committed to identifying additional and novel biomarkers of AD, something that is being discussed as part of an upcoming continuing grant renewal. Other efforts may focus on increasing protocol harmonization (where possible) with other longitudinal efforts, such as Horizon 21 and DABNI. It will be important to be able to combine data, when possible, to create even larger cohorts that will allow us to answer critical questions related to AD biomarkers and symptoms among the DS adult population worldwide. Similarly, we hope to continue to compare and contrast ABC‐DS findings with other at‐risk populations, such as adults with autosomal dominant AD or with aging adults from the general population. ABC‐DS also remains an integral part of Alzheimer's Clinical Trial Consortium for Down Syndrome (TRC‐DS) and most ABC‐DS sites will be actively involved in offering a number of clinical trial options to families. Toward this end, the Consortium has also been discussing how best to share research findings with participants and their families. For example, enrollment in most AD prevention trials will require that a participant meet a prespecified cutoff for amyloid. ABC‐DS investigators and staff will need to develop best practices for sharing this information with families (if desired) along with discussion of the implications and treatment options. ABC‐DS continues to focus on recruitment of participants from underserved groups and will likely add additional sites that have a record of strong recruitment in this area.

Finally, the Consortium is actively supporting the submission of additional grant proposals (e.g., R01s, R21s) that would leverage ABC‐DS data and/or provide further research opportunities for currently enrolled ABC‐DS participants. We have already been successful in serving as the “parent grant” for nine other NIH‐funded projects and a number of additional grant proposals are currently under review that will utilize ABC‐DS findings and resources. The ABC‐DS has already had a significant impact on the design and selection of assessment tools to be used in many of the AD prevention trials for the DS population. It is positioned to continue to make substantial contributions to our understanding of AD in adults with DS and the identification of biomarkers of preclinical AD progression.

## CONFLICT OF INTEREST STATEMENT

The authors declare no conflicts of interest. Author disclosures are available in the Supporting information.

## CONSENT STATEMENT

Informed consent/assent from all participants or their legally authorized representatives were obtained prior to inclusion.

## Supporting information



Supporting Information
